# Molecular characterization, clinical relevance and immune feature of m7G regulator genes across 33 cancer types

**DOI:** 10.3389/fgene.2022.981567

**Published:** 2022-08-25

**Authors:** Zhanzhan Li, Yanyan Li, Lin Shen, Liangfang Shen, Na Li

**Affiliations:** ^1^ Department of Oncology, Xiangya Hospital, Central South University, Changsha, China; ^2^ Department of Nursing, Xiangya Hospital, Central South University, Changsha, China; ^3^ National Clinical Research Center for Geriatric Disorders, Xiangya Hospital, Central South University, Changsha, China

**Keywords:** N^7^ -methylguanosine (m7G), RNA modification, methylation, immunotherapy, cancer

## Abstract

Over 170 RNA modifications have been identified after transcriptions, involving in regulation of RNA splicing, processing, translation and decay. Growing evidence has unmasked the crucial role of N^6^-methyladenosine (m6A) in cancer development and progression, while, as a relative newly found RNA modification, N^7^-methylguanosine (m7G) is also certified to participate in tumorigenesis *via* different catalytic machinery from that of m6A. However, system analysis on m7G RNA modification-related regulator genes is lack. In this study, we first investigated the genetic alteration of m7G related regulator genes in 33 cancers, and found mRNA expression levels of most regulator genes were positively correlated with copy number variation (CNV) and negatively correlated with methylation in most cancers. We built a m7G RNA modification model based on the enrichment of the regulator gene scores to evaluate the m7G modification levels in 33 cancers, and investigated the connections of m7G scores to clinical outcomes. Furthermore, we paid close attention to the role of m7G in immunology due to the widely used immune checkpoint blockade therapy. Our results showed the higher m7G scores related to immunosuppression of tumor cells. Further confirmation with phase 3 clinical data with application of anti-PDL1/PDL indicated the impact of m7G modification level on immunotherapy effect. Relevance of m7G regulator genes and drug sensitivity was also evaluated to provide a better treatment choice when treating cancers. In summary, our study uncovered the profile of m7G RNA modification through various cancers, and figured out the connection of m7G modification levels with therapeutical outcomes, providing potential better options of cancer treatment.

## Introduction

With the advancement of next-generation sequencing technologies, over 170 RNA modifications have been identified to be widely spread in eukaryotes and prokaryotes, occurring in ribosomal RNA (rRNA), messenger RNA (mRNA), transfer RNA (tRNA), noncoding small RNA (sncRNA) and long-chain non-coding RNA (lncRNA) (Zhou et al., 2020; Wiener and Schwartz, 2021). One of the representative modifications is RNA methylation which has been proven to participate in the cancer development and progression (Zhang et al., 2021). In recent years, there are growing number of reports revealed the biological functions of N^6^-methyladenosine (m6A) which is the most abundant modification in mRNA (Jiang et al., 2021). Benefit from the sustained effort made in understanding m6A, some other RNA modifications have been identified as well on the base of innovated mapping tools, such as N^7^-methylguanosine (m7G). m7G is often found at nucleotide 46 of tRNA variable loop, which is generated by tRNA (m^7^G46) methyltransferase (Tomikawa, 2018), and it is also found in other RNA species, such as mRNA, rRNA, etc.

Dynamic RNA methylation modification is balanced by methyltransferases (writers) and demethylases (erasers), and the methylation sites can be recognized by specific RNA-binding proteins (readers) that regulating splicing, stability, translation efficiency and structure of RNA (Rong et al., 2021). Plenty of proteins have been found to execute the corresponding functions in regard to m6A modification, however, limited reports revealed the mechanisms underlying m7G regulation. In human, methyltransferase like 1-WD repeat domain 4 (METTL1-WDR4) complex has been discovered to regulate the m7G modification process (Teng et al., 2021). Mutation in *WDR4* gene causes a distinct form of microcephalic primordial dwarfism (Shaheen et al., 2015). METTL1 knockout in mouse embryonic stem cells (mESCs) influence the translation of cell cycle genes and genes contributed to brain abnormalities, METTL1 or WDR4 knockout impairs the ESC self-renewal and differentiation (Lin et al., 2018). The latest research has revealed the role of METTL1-WDR4 complex in cancers. METTL1 and WDR4 are upregulated in hepatocellular carcinoma (HCC) and intrahepatic cholangiocarcinoma (ICC), related with poor prognosis. METTL1 mediated m7G tRNA modification promotes hepatocarcinogenesis, impaired m7G tRNA modification inhibits ICC tumorigenesis (Chen et al., 2021; Dai et al., 2021). Another report has pointed out METTL1 mediated tRNA modification, particularly Arg-TCT-4-1, increases the mRNA translation of growth-promoting proteins, drives oncogenic transformation (Orellana et al., 2021).

As one representative post-transcriptional RNA modification, m7G modification is of crucial importance in cancer development and progression (Luo et al., 2022). We believe that further understanding of mechanisms of m7G modification will contribute to the cancer treatment. In the study, we first analyzed the alteration status of m7G RNA modification-related regulator genes through 33 cancer types. Then, we used single sample gene set enrichment analysis (ssGSEA) method to calculate the “m7G score” on the basis of these regulator genes’ enrichments, representing the m7G RNA modification level. The correlation of m7G score and clinical outcome endpoints were analyzed in cancers, and we also investigated the relationship of m7G score and immunology. Besides, the correlation of m7G RNA modification-related regulator genes and drug sensitivity was studied. In summary, our results might provide a potential better therapeutic treatment alternative towards various cancer types.

## Methods

### Data source and differential expressed gene analysis

Gene expression RNAseq (HTSeq) of 33 cancer and normal tissues of patients, along with the corresponding clinical parameters were downloaded from University of California SANTA CRUZ (https://xenabrowser.net/datapages/). Up to 29 m7G RNA modification-related regulator genes (Li et al., 2022) in 33 cancers were subjected to differential expression analysis using “limma” R package. The significant differential expression was defined when |logFC|>1, *p* < 0.05. The list of genes and cancer types was in Supplementary Tables S1, S2.

### Somatic copy number alterations, mutation and methylation analysis

Single nucleotide variation (SNV), copy number variation (CNV) and methylation of m7G regulator genes in various cancers were analyzed using Gene Set Cancer Analysis (GSCA) database (http://bioinfo.life.hust.edu.cn/GSCA/#/). Pearson’s correlation coefficient was applied to show the correlation of CNV or methylation with mRNA expression of the genes. Methylation difference between cancer and normal samples of regulator gents through various cancers was also evaluated using “limma” R package.

### Establishment the m7G modification level model

We calculated the m7G score using the ssGSEA method, which allows one to define an enrichment score that represents the degree of absolute enrichment of a gene set in each sample within a given data set.

For a given signature G of size NG and single sample S, of the data set of N genes, the genes are replaced by their ranks according their absolute expression from high to low: An enrichment score ES (G, S) is obtained by a sum (integration) of the difference between a weighted ECDF of the genes in the signature and the ECDF of the remaining genes PNG (Barbie et al., 2009):

$$ES\left(G,S\right)=\sum_{i=1}^{N}\left[P_{G}^{w}\left(G,S,\;i\right)-P_{N_{G}}\left(G,S,i\right)\right]$$

We completed the calculation using the R “GSEABase” packages.

### Survival analysis

The impaction of m7G on the survival prognosis of cancers was evaluated, including overall survival (OS), disease-specific survival (DSS), progression-free interval (PFI), and disease-free interval (DFI). Cox regression was utilized to reveal the hazard ratio of m7G scores in various cancers. Patients were divided into 2 groups with high and low m7G scores, Kaplan-Meier analysis survival curve was used to compare the survival probabilities between 2 groups. *p* < 0.05 was considered significant.

### Immune feature analysis

Immune-associated data were downloaded from Immune Cell Abundance Identifier (ImmuCellAI) database (http://bioinfo.life.hust.edu.cn/ImmuCellAI/#!/). Estimation of STromal and Immune cells in MAlignant Tumor tissues using Expression data (ESTIMATE) algorithm (Yoshihara et al., 2013) was used to analyze immune components and overall stroma. Pearson correlation coefficients was applied to reveal the association of m7G score and immune parameters (tumor purity, stromal score, immune score and ESTIMATE score) and immune cells (B cells, T cells, myeloid dendritic cells, endothelial cells, NK cells, macrophages, and immune cell subsets. The association of m7G score and some crucial pathways were evaluated, including immune related pathways (antigen processing machinery, immune checkpoint, CD8 T-effector), matrix/metastasis related pathways (EMT1, EMT2, EMT3, pan fibroblast TGF-β response signature), and DNA damage/repair related pathways (nucleotide excision repair, mismatch repair, DNA replication, base excision repair, DNA damage response). Impact of m7G modification level mediated in immunotherapy was investigated using the data downloaded from NCT02684006 KIRC (PMID: 32895571) (Motzer et al., 2020) and Checkmate KIRC (PMID: 32472114) (Braun et al., 2020) cohorts.

### Drug sensitivity analysis

The drug response data and genomic markers of sensitivity were downloaded from Genomics of Drug Sensitivity in Cancer (https://www.cancerrxgene.org/) and Cancer Therapeutics Response Portal (https://portals.broadinstitute.org/ctrp/). The association of m7G score and small molecule drugs were evaluated by Pearson correlation coefficients.

## Results

### Genetic alteration portfolio of m7G RNA modification-related regulator genes in cancers

**FIGURE 1:**
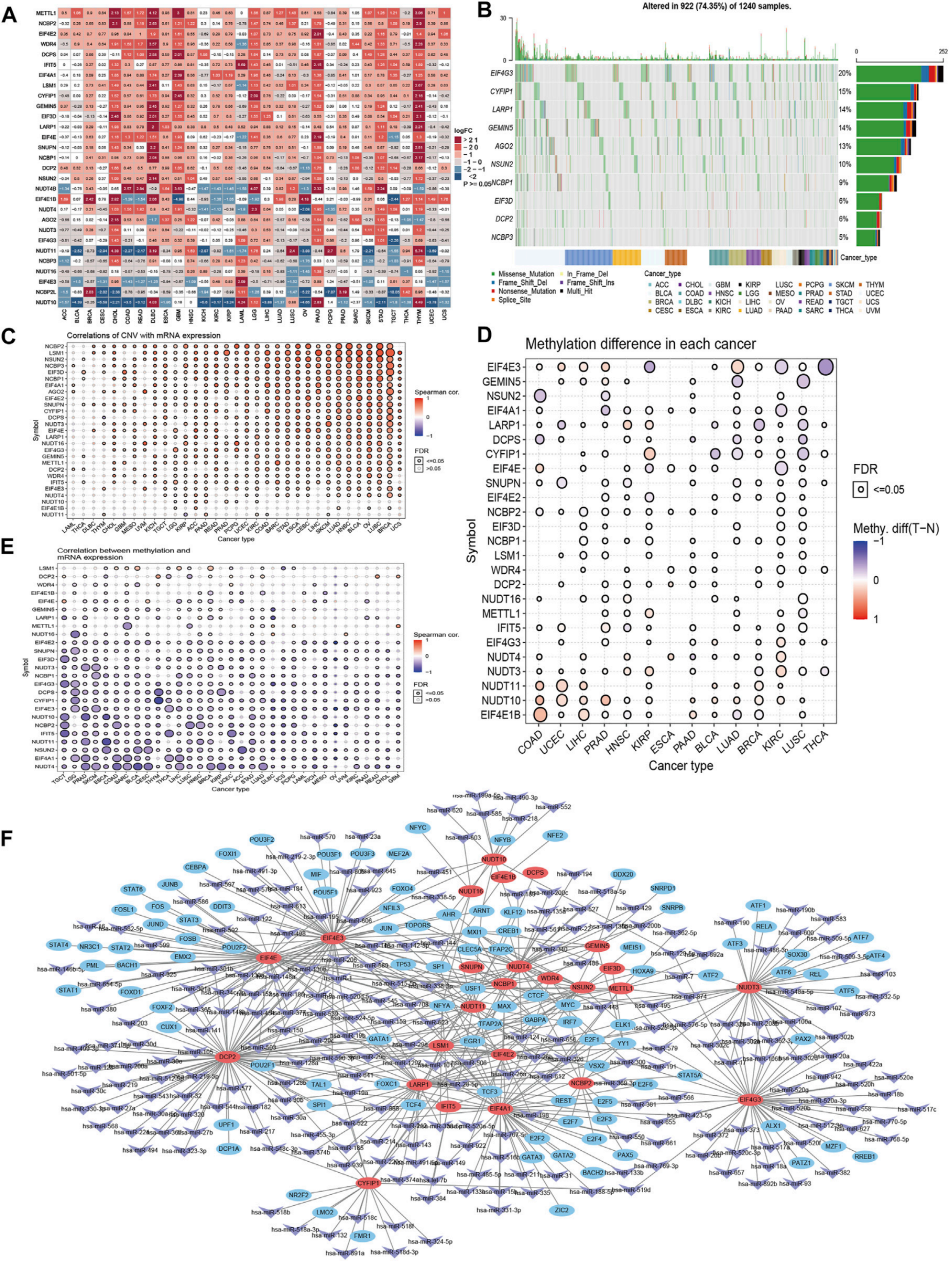
Genetic alteration portfolio of m7G RNA modification-related regulator genes in cancers. **(A)** Differential expression of 29 m7G related regulator genes in 33 cancers. **(B)** Summary of top 10 gene variations in selected cancers. **(C)** Correlation of regulator gene expression and CNV. **(D)** Methylation level of regulator genes varied in each cancer. **(E)** Correlation of regulator gene expression and methylation level. **(F)** Network of predictive miRNAs and transcription factors mediated in regulation of m7G related regulator genes.

In this study, we assessed the expression of 29 potential regulator genes involved in m7G RNA modification in 33 different cancer tissues and corresponding normal tissues. We found that more differential expressed genes (≥20 out of 29 regulator genes, |logFC|>1, *p* < 0.05) were harbored in Cholangio Carcinoma (CHOL), Lymphoid Neoplasm Diffuse Large B-cell Lymphoma (DLBC), Pancreatic Adenocarcinoma (PAAD) and Thymoma (THYM) (Figure 1A), and 5 main differential expressed regulator genes (*EIF4E1B, METTL1, NUDT11, NUDT10* and *NUDT4B*) were distributed in more than 15 cancer types (|logFC|>1, *p* < 0.05). Then, we examined these gene alterations, including gene sequence and structural variants, among cancers. About 74.35% (922 out of 1,240) samples contained the sequence variants, while missense mutation seemed more frequent, with most contribution from 5 genes (*EIF4G3, CYFIP1, LARP1, GEMIN5* and *AGO2*) (Figure 1B). Copy number variation (CNV), as one of the structural variants, was proven to be closely linked to cancers by affecting gene expression with dosage effect (Hovhannisyan et al., 2019). We discovered that mRNA expression levels of most regulator genes were correlated with CNV (Figure 1C). For instance, the CNV of *NCBP2* positively related to mRNA expression in nearly 30 cancer types. Nuclear cap-binding protein subunit 2 protein (NCBP2) is one component of cap-binding complex which binds to the m7G capped RNA, participate in RNA splicing, transportation and RNA decay (Dou et al., 2020). We further described percentage of heterozygous and homozygous variants of CNV including amplification and deletion in 33 cancers (Supplementary Figures S1A–C). The result showed the main CNV of regulator genes were heterozygous amplification (Hete Amp) or heterozygous deletion (Hete Del). Moreover, we assessed methylation levels of these regulator genes through 14 cancers tissues and corresponding normal tissues, and realized it is complicated as they might exhibit higher methylation level in one cancer type and lower level in another, or none differences were discovered between cancer and normal tissues (Figure 1D). However, negative correlation between methylation and mRNA expression of regulator genes were seen in most of cancer types (Figure 1E), indicating the expression of regulator genes might be regulated by the DNA methylation. Considering the essential role of microRNA (miRNA) and transcription factor acting on gene regulation, we described the network of predictive miRNAs and transcription factors mediated in m7G regulator gene regulations (Figure 1F). Majority genes (25 out of 29 regulator genes) were included in this complicated regulatory network, while some could be targeted with higher frequency, such as *EIF4A1, EIF4E, EIF4E2, EIF4E3, EIF4G3, DCP2, LARP1, NUDT3, NUDT4*, etc.

### Establish modeling of the m7G modification level among cancers

**FIGURE 2:**
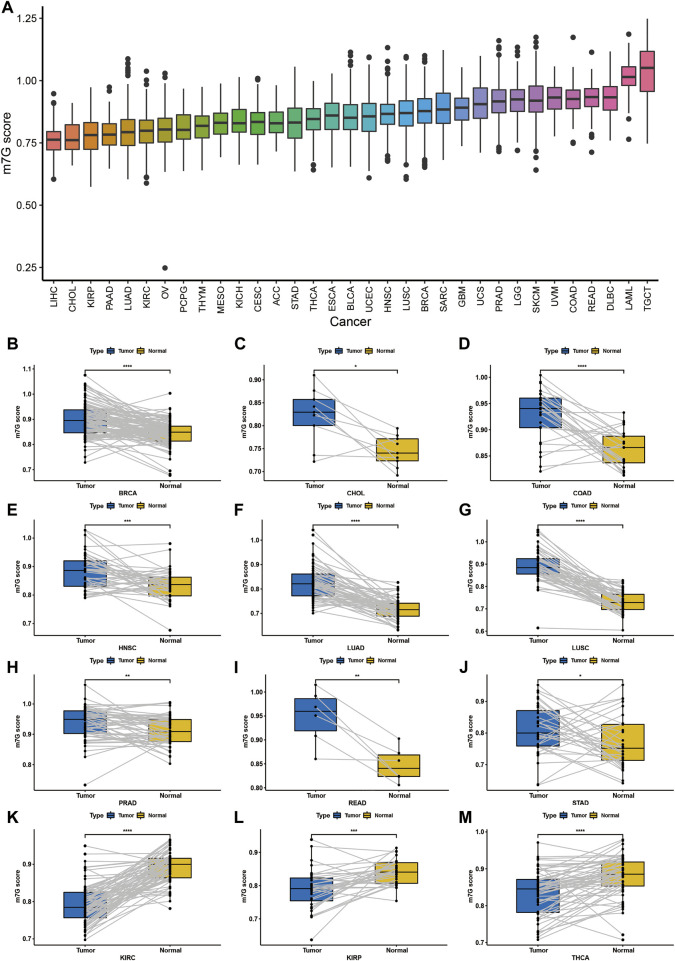
Establish modeling of the m7G modification level among cancers. **(A)** m7G modification levels in 33 cancers. **(B–J)** m7G modification levels were elevated in tumor tissues (BRCA, CHOL, COAD, HNSC, LUAD, LUSC, PRAD, READ and STAD) than the corresponding normal tissues. **(K–M)** m7G modification levels were decreased in tumor tissues (KIRC, KIRP and THCA) than the corresponding normal tissues.

In order to reveal the potential function of m7G RNA modification involved in the tumor development and biological processes, we used single-sample GSEA to calculate the m7G scores representing m7G modification levels in 33 cancer types based on the enrichment scores. Obviously, Liver Hepatocellular Carcinoma (LIHC) got the lowest m7G score while Testicular Germ Cell Tumors (TGCT) got the highest score (Figure 2A).

Considering RNA methylation modifications shared the similar catalytic mechanism that was balanced by methyltransferases and demethylases, we evaluated the relationship of m7G scores and m6A regulator gene (Zhang et al., 2020a; Zhao et al., 2021a) expressions in various cancer types by means of Spearman correlation. m6A regulator genes were shown to play important oncogenic roles in cancers (Arumugam et al., 2021). As shown in Supplementary Figure S2, most m6A regulator genes showed positive correlation with m7G scores, especially *METTL14* and *HNRNPC*. While in LIHC and TGCT, with lowest and highest m7G score respectively, majority m6A regulator genes exhibited positive correlations with m7G scores and 2 or 3 genes exhibited negative correlations with m7G scores. In OV, all m6A regulator gene expressions showed significant positive correlations with m7G score value that ranked in between according to our model. We believed the m6A regulator genes behaved differently from our m7G regulator genes in different cancers.

Furthermore, we investigated the m7G scores between tumor and normal tissues, and we found that the m7G modification level was significant elevated in Breast invasive carcinoma (BRCA), CHOL, Colon adenocarcinoma (COAD), Head and Neck squamous cell carcinoma (HNSC), Lung adenocarcinoma (LUAD), Lung squamous cell carcinoma (LUSC), Prostate adenocarcinoma (PRAD), Rectum adenocarcinoma (READ) and Stomach adenocarcinoma (STAD) (Figures 2B–J), while significant decreased in Kidney renal clear cell carcinoma (KIRC), Kidney renal papillary cell carcinoma (KIRP) and Thyroid carcinoma (THCA) (Figures 2K–M).

**FIGURE 3:**
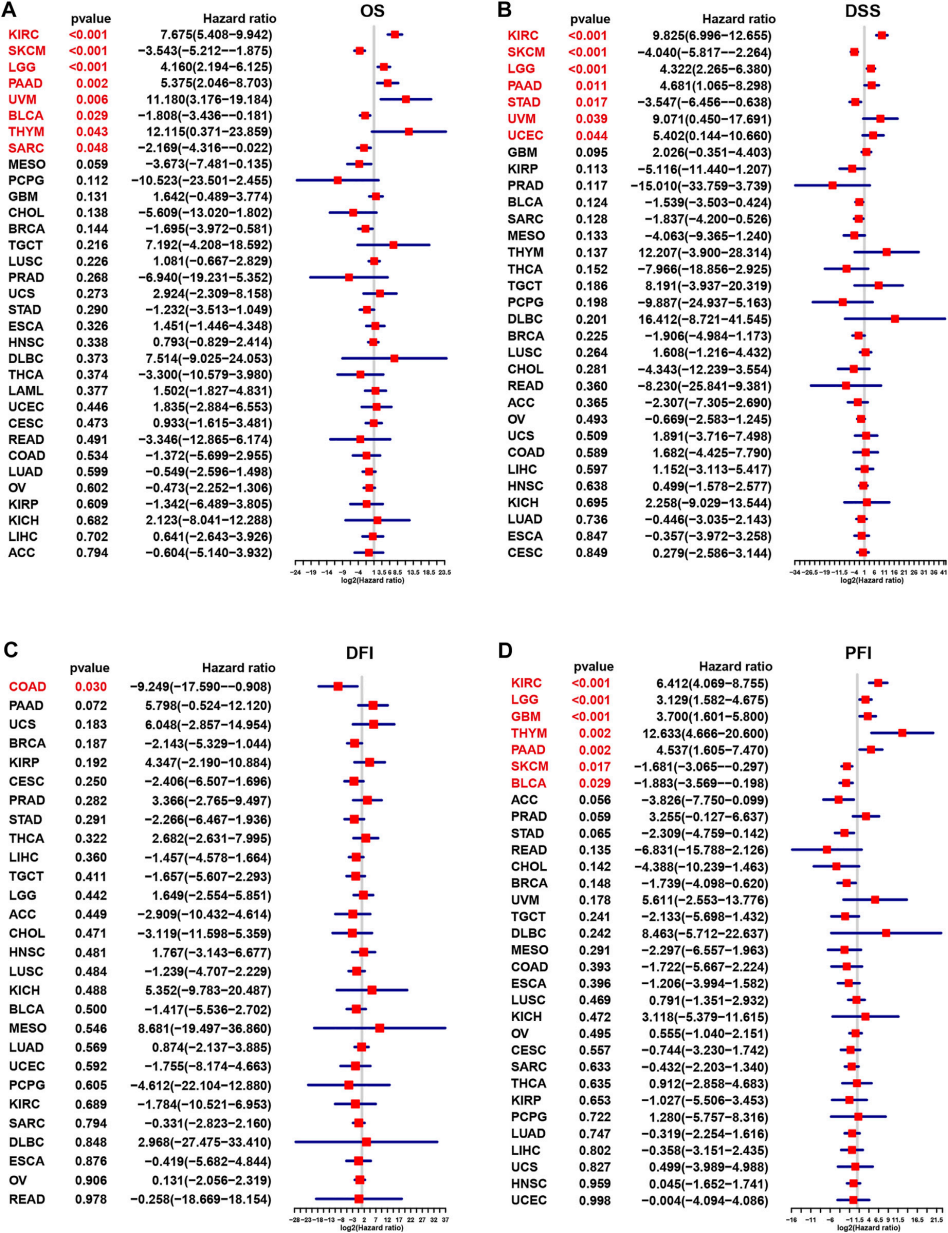
Correlation of m7G modification level with prognosis in each cancer based on Cox regression.**(A)** Overall survival. **(B)** Disease-specific survival. **(C)** Progression-free interval. **(D)** Disease-free interval.

Next, univariate Cox regression was utilized to discover the relationship of m7G modification level and clinical outcome endpoints. The m7G score was associated with OS (Figure 3A) in 8 cancer types, that were KIRC (*p* < 0.001), Skin Cutaneous Melanoma (SKCM, *p* < 0.001), Brain Lower Grade Glioma (LGG, *p* < 0.001), PAAD (*p* = 0.002), Uveal Melanoma (UVM, *p* = 0.006), Bladder Urothelial Carcinoma (BLCA, *p* = 0.029), THYM (*p* = 0.043) and Sarcoma (SARC, *p* = 0.048). While the m7G score was associated with DSS (Figure 3B) in 7 cancer types, KIRC (*p* < 0.001), SKCM (*p* < 0.001), LGG (*p* < 0.001), PAAD (*p* = 0.011), STAD (*p* = 0.017), UVM (*p* = 0.039) and Uterine Corpus Endometrial Carcinoma (UCEC, *p* = 0.044). As to DFI (Figure 3C), only 1 cancer type were screened out based on the m7G score, that was COAD (*p* = 0.03), and m7G score correlated with PFI (Figure 3D) in 7 cancer types, that were KIRC (*p* < 0.001), LGG (*p* < 0.001), Glioblastoma multiforme (GBM, *p* < 0.001), THYM (*p* = 0.002), PAAD (*p* = 0.002), SKCM (*p* = 0.017) and BLCA (*p* = 0.029). In addition, survival analyses were conducted in 33 cancer types. The significant correlation of m7G scores and OS were found in 7 cancer types (BRCA, HNSC, KIRC, PAAD, PCPG, READ and SARC) (Supplementary Figures S3A–G), m7G scores and DFI in 5 cancer types (HNSC, LGG, PCPG, PADD, and STAD) (Supplementary Figures S4A–E), m7G scores and DSS in SARC (Supplementary Figure S4F), m7G scores and PFI in 5 cancer types (COAD, LIHC, BLCA, PAAD, PCPG) (Supplementary Figures S4G–K).

### Correlation of m7G modification level and cell signaling pathways in 33 cancers

**FIGURE 4:**
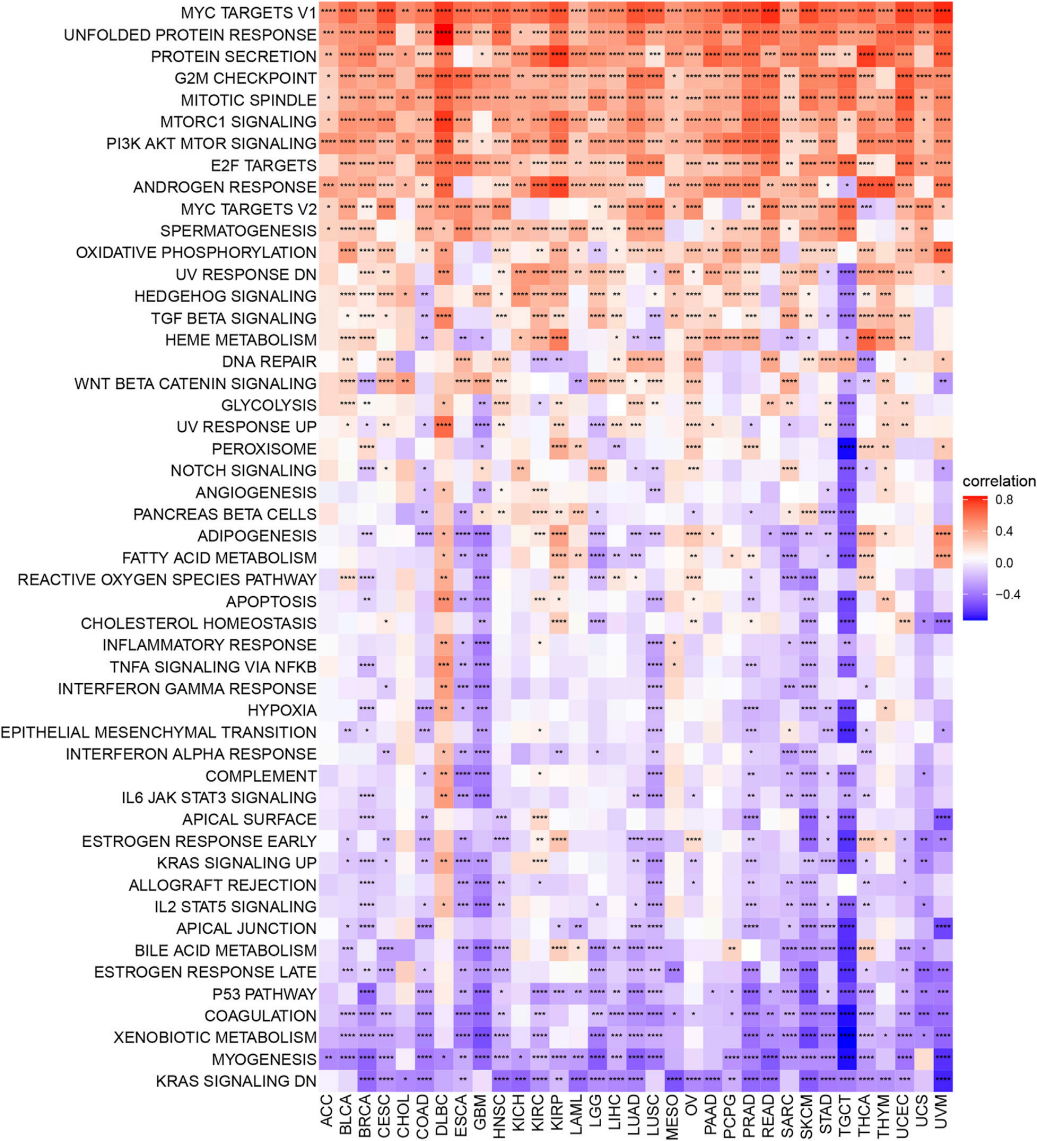
Correlation of m7G modification level and cell signaling pathways in 33 cancers.

To better understand the relationship of m7G modification regulation and various cell signaling pathways among cancers, we calculated Spearman correlation coefficients to exhibit the relevance of the m7G scores and various pathways scored by GSVA. As shown in Figure 4, a subgroup of genes regulated by Myc-version 1 (Myc targets V1), unfolded protein response, protein secretion, G2M checkpoint, mitotic spindle, MTORC1 signaling, and PI3K AKT MTOR signaling were positively corelated with m7G score in most cancer types (≥30 cancers), while genes down-regulated by KRAS activation (KRAS signaling DN), myogenesis, xenobiotic metabolism, coagulation and p53 pathway were negatively corelated with m7G score in some cancers (≥23 cancers).

### Correlation of m7G modification level and immunology in 33 cancers

**FIGURE 5:**
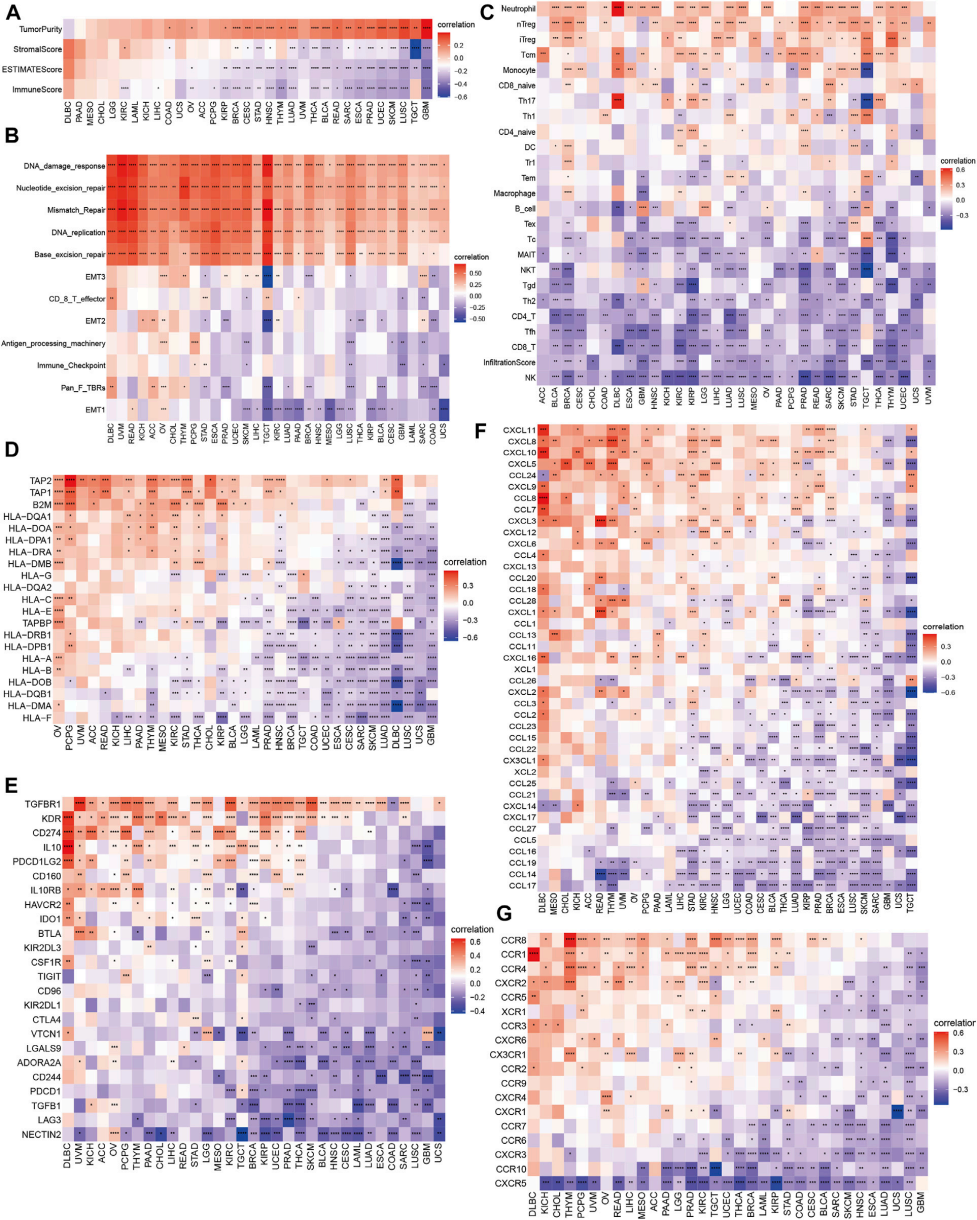
Correlation of m7G modification level and immune features in 33 cancers. **(A)** Tumor microenvironment. **(B)** Immune related pathways, matrix/metastasis related pathways and DNA damage/repair related pathways. **(C)** Immune cell subsets. **(D)** Major histocompatibility complex-related genes. **(E)** Immune suppression genes. **(F,G)** Chemokines and their receptors.

Growing evidence indicates the pivotal role of tumor microenvironment (TME) in tumor treatment, we investigated the correlation of m7G scores and TME in 33 cancers. As shown in Figure 5A, m7G scores were positively correlated with tumor purity in majority cancer types, excluding DLBC and PAAD. However, both positive and negative correlation were observed between m7G scores and stromal/immune/ESTIMATE scores through various cancer types. Therefore, we evaluated several biological processes connected to TME (Zeng et al., 2019), including immune related pathways (antigen processing machinery, immune checkpoint, CD8 T-effector), matrix/metastasis related pathways (EMT1, EMT2, EMT3, pan fibroblast TGF-β response signature), and DNA damage/repair related pathways (nucleotide excision repair, mismatch repair, DNA replication, base excision repair, DNA damage response). We found m7G scores were positively related to DNA damage/repair related pathways (Figure 5B). Relevance of m7G scores and immune cell infiltration were also assessed. Of note, m7G scores were negatively correlated with CD4 T cells, CD8 T cells and NK cells but positively correlated with Treg cells in most of cancer types (Figure 5C), while the relevance with naïve CD4 and CD8 cells were not obviously seen through cancers. We speculated that higher m7G scores might relate to immunosuppression in tumor cells.

**FIGURE 6:**
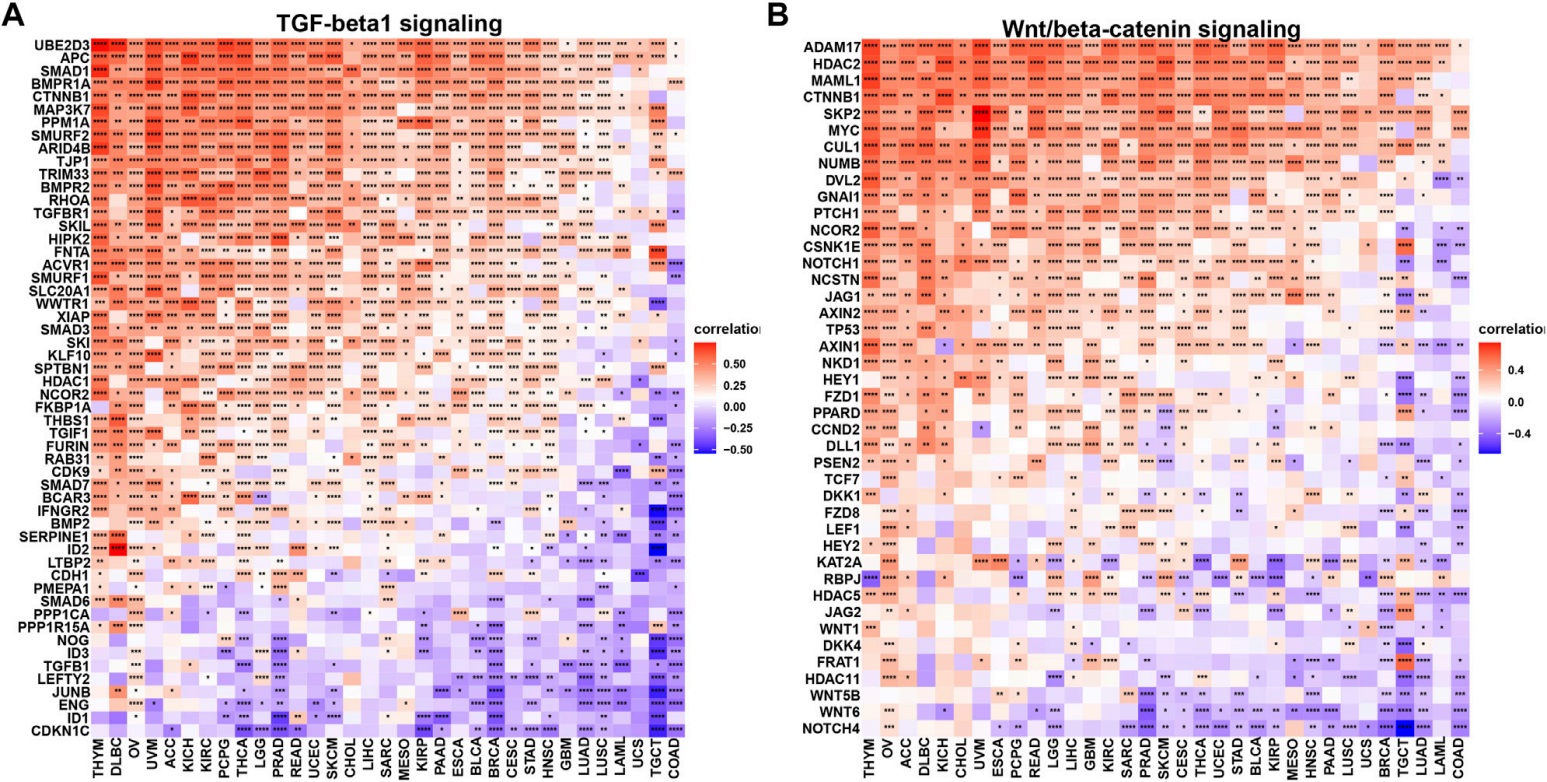
Correlation of m7G modification level and TGF-β1 and Wnt/β-catenin signaling pathways.**(A)** TGF-β1 signaling pathway. **(B)** Wnt/β-catenin signaling pathway.

Moreover, we elaborated the association of m7G scores with MHC genes (Figure 5D), immuno-suppressive genes (Figure 5E), chemokines (Figure 5F) and chemokine receptors (Figure 5G). It is notable that most immuno-suppressive genes were negatively related to m7G scores through 33 cancers, such as famous drug targets PD1 (gene: *PDCD1*, ≥30 cancers), CTLA4 (gene: *CTLA4*, ≥27 cancers) and the next immune checkpoint receptor LAG3 (Ruffo et al., 2019) (gene: *LAG3*, ≥27 cancers). Considering the important role of TGF-β1 and Wnt/β-catenin signaling modulating anticancer immune response (Yoshimura and Muto, 2011; Sheng et al., 2015; Pai et al., 2017; Takeuchi et al., 2021), we investigated the correlations of m7G scores and genes involved in those two signaling pathways (Figures 6A,B). *UBE2D3, APC, SMAD1, BMPR1A, CTNNB1*, and *MAP3K7* in TGF-β1 signaling pathway, showed positive correlation with m7G scores through most cancer types (≥30 cancers), while *ADAM17, HDAC2, MAML1, CTNNB1, SKP2, MYC* and *CUL1* in Wnt/β-catenin signaling positively correlated with m7G scores among most cancer types (≥30 cancers).

**FIGURE 7:**
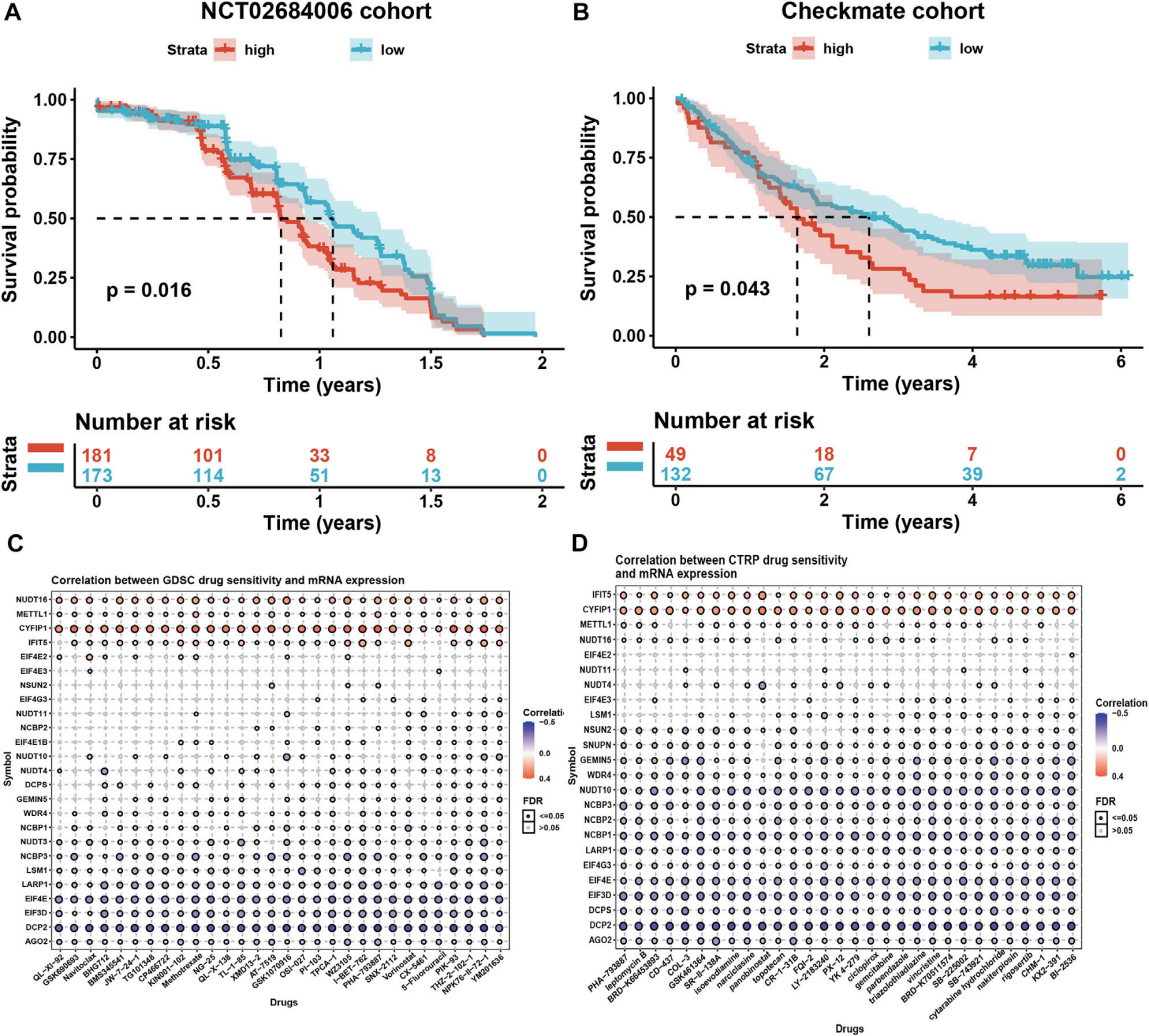
Associations of m7G modification level and immunotherapy and drug sensitivity. **(A)** High m7G modification level corelated to the low OS in advanced RCC patients with anti-PDL1 antibody treatment. **(B)** High m7G modification level corelated to the low OS in advanced clear cell RCC patients with anti-PD1 antibody treatment. **(C,D)** Correlation of m7G modification-related regulator gene expressions and drug sensitivity using GDSC and CTRP datasets.

Higher m7G scores seemed to be associated with inhibited immune status in cancer tissues, we wondered if the m7G modification level would affect the outcomes of the immunotherapy using the phase 3 clinical data of anti-PDL1 antibody Avelumab (NCT02684006) in advanced renal cell carcinoma (RCC) (Motzer et al., 2020). The result showed that the high m7G modification level corelated to the low OS in advanced RCC patients (*p* = 0.016, Figure 7A). Another data of prospective clinical trials evaluating PD1 antibody Nivolumab in advanced clear cell RCC (Braun et al., 2020) were analyzed, and the result also showed the negative correlation of m7G scores and OS (Figure 7B). In conclusion, we reckoned that m7G modification level might have impact on the immunotherapy effect.

### mRNA expression level of m7G modification-related regulators corelated with drug sensitivity

To further discuss the functions of m7G modification level in cancer treatment, we analyzed the correlation of mRNA expression level of m7G modification-related regulators and drug sensitivity using GDSC and CTRP datasets (Figures 7C,D). Top 30 compounds were selected towards regulator genes (|r|> 0.3) in pan-cancer. We noticed a small portion of regulator genes positively related to majority of compounds, such as *NUDT16* (related to 30 cancer drugs in GDSC, 21 in CTRP), *METTL1* (related to 30 cancer drugs in GDSC, 22 in CTRP), *CYFIP1* (related to 30 cancer drugs in GDSC, 30 in CTRP), and *IFIT5* (related to 26 cancer drugs in GDSC, 30 in CTRP), indicating the patients with higher mRNA expression level of these regulator genes might exhibit drug-resistance when treated with these compounds. However, more regulator genes negatively related to those compounds, like *AGO2, DCP2, EIF3D, EIF4E, LARP1,* and *NCBP3* (related to 30 cancer drugs both in GDSC and CTRP), indicating the patients with higher mRNA expression level of these regulator genes might sensitive to these compounds.

## Discussion

Nowadays, it is widely accepted the DNA and RNA modification both play an important role in regulating gene expression patterns and influence cancer development. DNA methylation (Wang et al., 2013) is a common epigenetic modification, controlling gene expression under certain environment (Bauer et al., 2016). Mechanisms underlying epigenetic modification has been well established (Gu et al., 2015; Blanco et al., 2021; Chu et al., 2022), however, the pivotal progress concerning RNA modification is less known. “Epitranscriptomics” was brought up recently referring to the post-transcriptional RNA modification, including m6A, pseudouridine (Ψ), 5-methylcytidine (m5C), N1- methyladenosine (m1A), N4-acetylcytidine (ac4C), ribose methylations (Nm) and N7- methylguanosine (m7G) (Wiener and Schwartz, 2021). m6A RNA methylation has been proven to regulate hematopoietic/central nervous system/reproductive system development, and malfunction of m6A RNA methylation corelates with various cancers (He et al., 2019; Gu et al., 2020; Huang et al., 2020; Melstrom and Chen, 2020; Jiang et al., 2021) in the last decade. On the contrary, the role of m7G RNA modification is revealed recently because of the limitation of detection methods in the past years.

m7G RNA methylation is found in tRNA, mRNA and microRNA (Pandolfini et al., 2019; Zhao et al., 2021b; Chen et al., 2021; Dai et al., 2021), while less knowledge has been gained with respect to the catalytic machinery. The most well characterized m7G regulator is METTL1-WDR4 complex, with impact on cancer development. In this study, we investigated the molecular characterization of m7G RNA modification related regulator genes. We found 5 primary regulator genes (*EIF4E1B, METTL1, NUDT11, NUDT10* and *NUDT4B*) were differential expressed in more than 15 cancer types. eIF4E1b (eIF4E family member 1b), recognizing and binding m7G-containing mRNA cap, is discovered highly expressed in mouse, *Xenopus* and zebrafish oocytes (Kubacka et al., 2015). eIF4E is overexpressed in some human cancers, involving in the mitosis, embryogenesis and apoptosis processes (Mamane et al., 2004). The evidence of METTL1-mediated cancer development is solid. *NUDT11* has been mentioned in prostate cancer pathogenesis (Grisanzio et al., 2012). *NUDT10* and *NUDT11* encode identical proteins with deferent expression patterns (Hua et al., 2003). Information about *NUDT4B* is scarce. Anyways, our study indicated the potential impact of these regulator genes working *via* the m7G RNA modification pathway, but further experiment verifications are necessary.

In order to give a general idea of m7G modification levels in various cancers, we built an estimated model using single-sample GSEA through 33 various cancers, and we found that the m7G modification level was significant elevated in BRCA, CHOL, COAD, HNSC, LUAD, LUSC, PRAD, READ, and STAD, while significant decreased in KIRC, KIRP and THCA. In addition, we adopted univariate Cox regression to discover the relationship of m7G modification level and clinical outcome endpoints. The results showed m7G score was associated with OS in KIRC, SKCM, LGG, PAAD, UVM, BLCA, THYM, and SARC. Moreover, we analyzed the relevance of m7G scores and key pathways, as well as the relationship of m7G scores and immune infiltration level. Accumulating evidences indicate that RNA methylation regulation plays an important role in human diseases and cancers (Luo et al., 2022), especially relates to tumor immunity (Zhang et al., 2021). Deletion of m6A “writer” protein METTL3 in mouse T cells will disrupt T cell homeostasis and differentiation (Li et al., 2017). Deletion of m6A binding protein YTHDF1 in mouse show an elevated antigen-specific CD8 T cell anti-tumor response (Han et al., 2019). Follicular helper T (Tfh) cells are a subset of CD4 T cells, helping B cells producing antibodies. The initiation and development of Tfh cells needs ICOS, whose expression could be inhibited by m6A modification catalyzed by METTL3 and METTL14 (Zhu et al., 2019). METTL3 knockout decreases the m6A methylation of SOCS family, and increases the SOCS expression that regulates the inhibitory function of Tregs (Tong et al., 2018). Our study disclosed that m7G scores were negatively correlated with CD4 T cells, CD8 T cells and NK cells but positively correlated with Treg cells in most of cancer types, while the concrete mechanisms are still in a mist. Deletion of m6A demethylase Alkbh5 increases m6A density near splice sites, leads to aberrant RNA splicing, sensitizes tumors to anti-PD1 therapy (Li et al., 2020). Deficiency of another m6A demethylase FTO decreases the mRNA and protein expression level of PDL1 in colon cancer cells (Tsuruta et al., 2020). Besides, some other bioinformatic analyses build risk models based on the m6A modification related regulator genes, attempt to evaluate the immunotherapy efficacy. For example, a study on gastric cancer reports that patients with low m6A score demonstrate a better survival benefit in STAD cohort and exhibit a markedly prolonged survival to checkpoint blockade therapy in both anti-PD-L1 cohort (IMvigor210) and anti-PD-1 cohort (GSE78220) (Zhang et al., 2020b). Hereon, we didn’t see the correlation of m7G score with OS in STAD, but our results indicated that m7G scores were negatively related to many immuno-suppressive genes like *PDCD1, CTLA4* and *LAG3* through various cancers, and we verified with clinic data showing high m7G scores corelated to the low OS in advanced and advanced clear cell RCC when using immune checkpoint blockade therapy. In view of the pivotal impact of RNA methylation on tumor immunity, it would be of interest to evaluate the immunotherapy outcome from the angle of RNA methylation. However, we realized some of the m7G regulator genes could have other functions in various cancers, and other RNA methylations might share the similar mechanism, like m6A, m5C, m1A. Further experiments are needed to verified the mechanisms of m7G modification.

Hereon, our results indicated that m7G scores were negatively related to many immuno-suppressive genes like *PDCD1, CTLA4* and *LAG3* through various cancers, and we verified with clinic data showing high m7G scores corelated to the low OS in advanced and advanced clear cell RCC when using Immune checkpoint blockade therapy. In view of the pivotal impact of RNA methylation on tumor immunity, it would be of interest to evaluate the immunotherapy outcome from the angle of RNA methylation.

In conclusion, we have elaborated genetic alteration of m7G RNA modification-related regulator genes, and established a model on the gene enrichment scores to reveal the m7G RNA modification levels throughout 33 various cancers. Correlations of m7G scores and OS, DSS, DFI and PFI of patients were uncovered in cancers. Of note, higher m7G scores seemed to be associated with inhibited immune status in cancers, hopefully that would provide some advices on the immunotherapy choice when treating different cancers. In addition, we evaluated the connection of m7G scores and drug sensitivity. Further work on the underlying mechanisms needs to be done to fully understand the biological functions of m7G RNA modification.

## Data Availability

The datasets presented in this study can be found in online repositories. The names of the repository/repositories and accession number(s) can be found in the article/Supplementary Material.
