# Supplementary figures and images for: Molecular characterization, clinical relevance and immune feature of m7G regulator genes across 33 cancer types
Source: Front Genet. 2022 Aug 25;13:981567. doi: 10.3389/fgene.2022.981567 (PMC9453236; doi:10.3389/fgene.2022.981567)

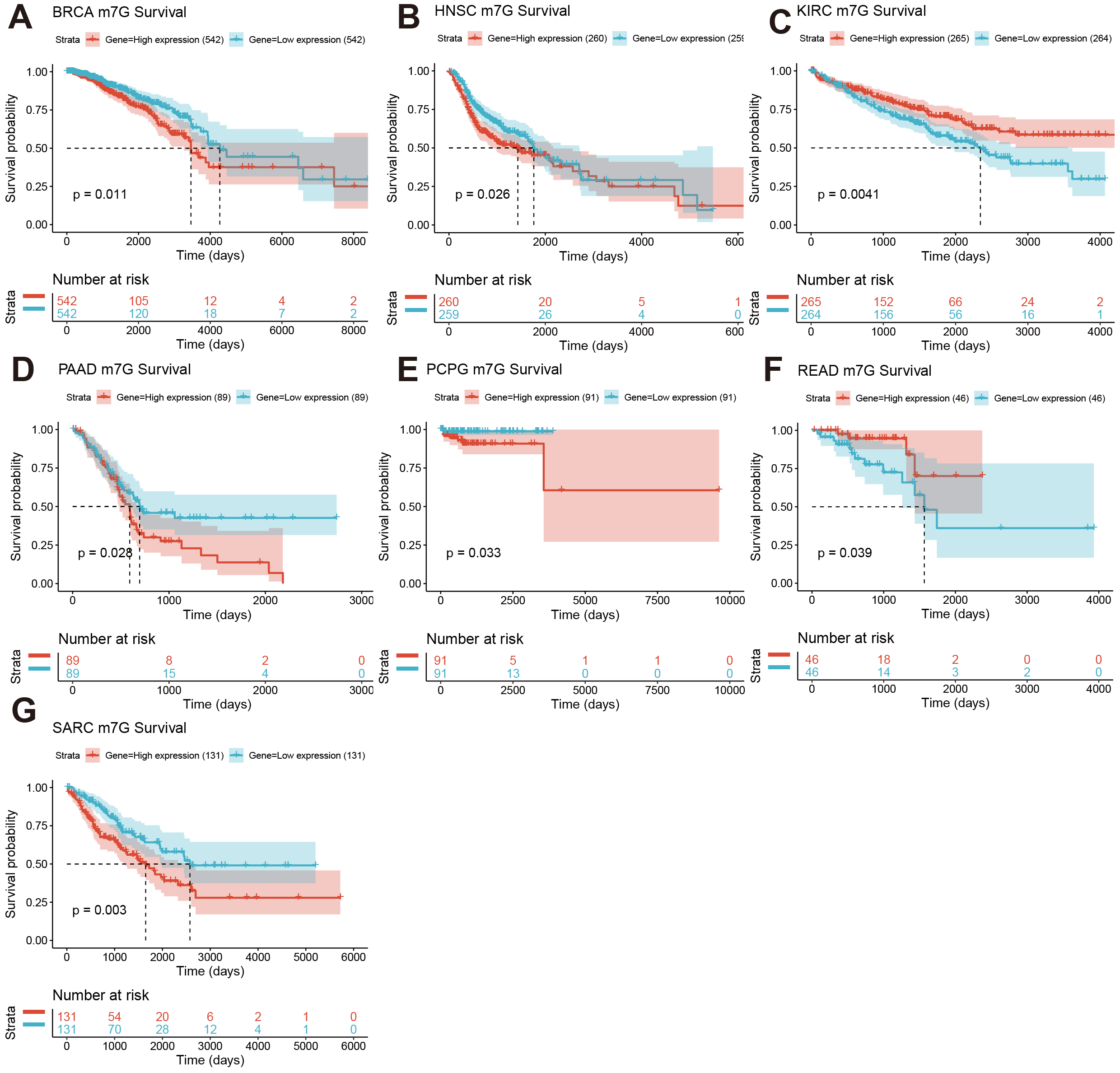

Supplement: Supplementary file 2 [file Image3.TIF]

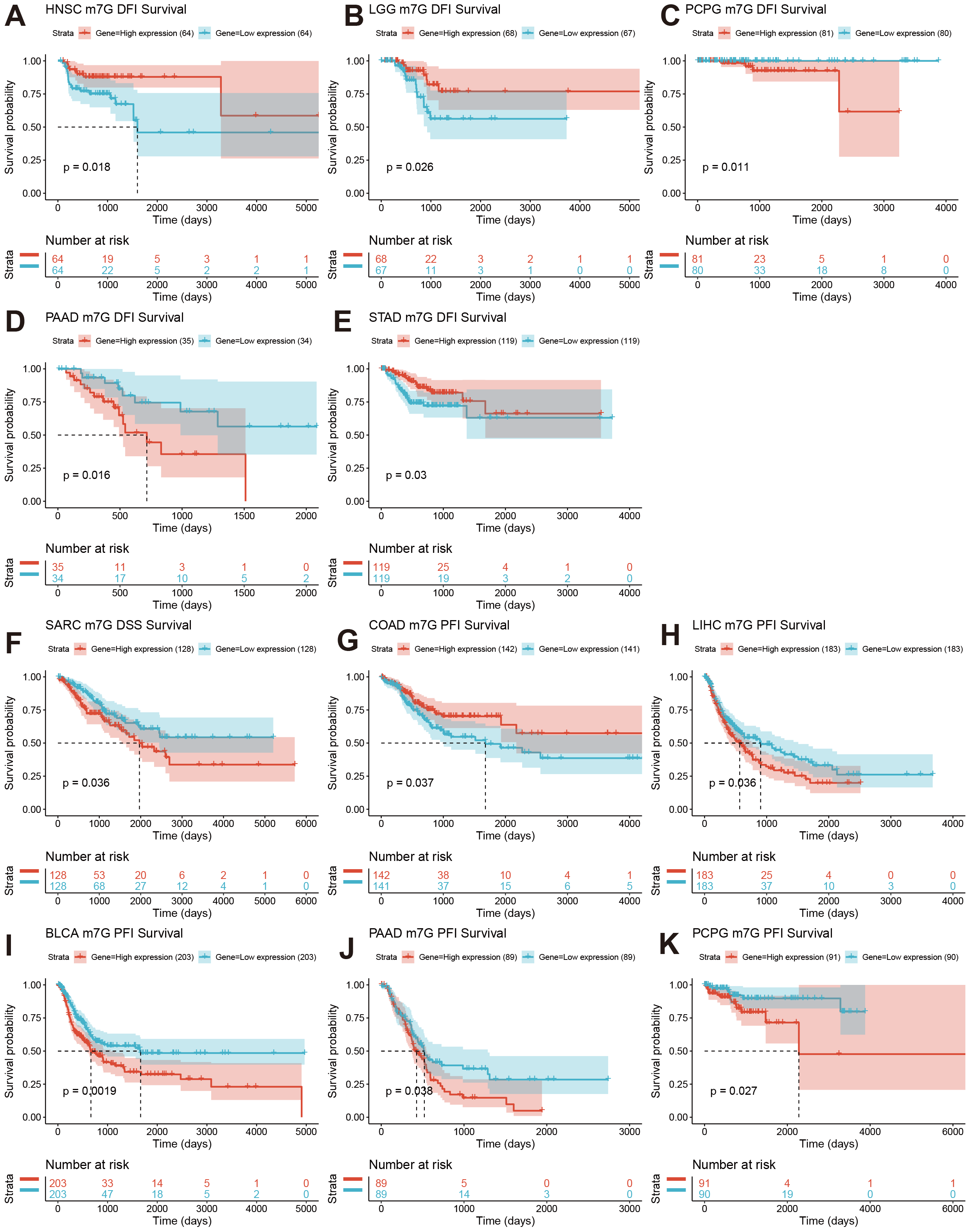

Supplement: Supplementary file 3 [file Image4.TIF]

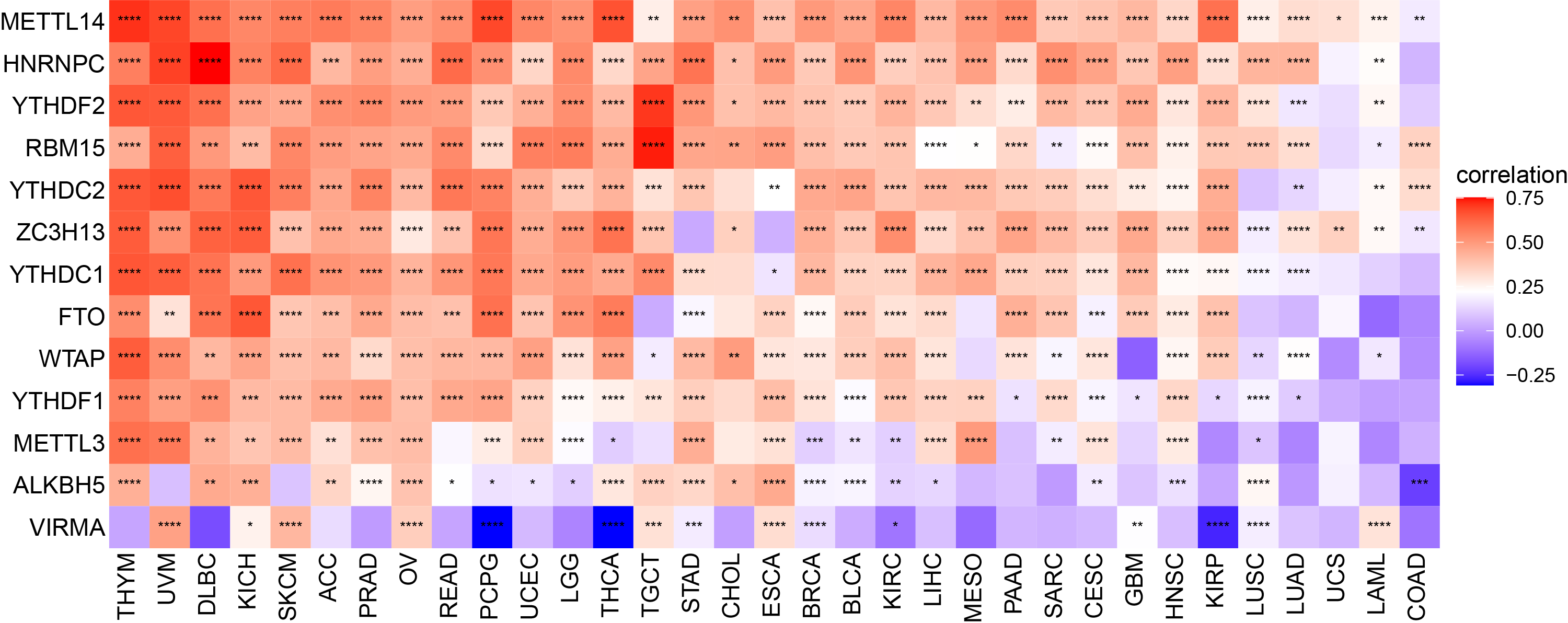

Supplement: Supplementary file 4 [file Image2.TIF]

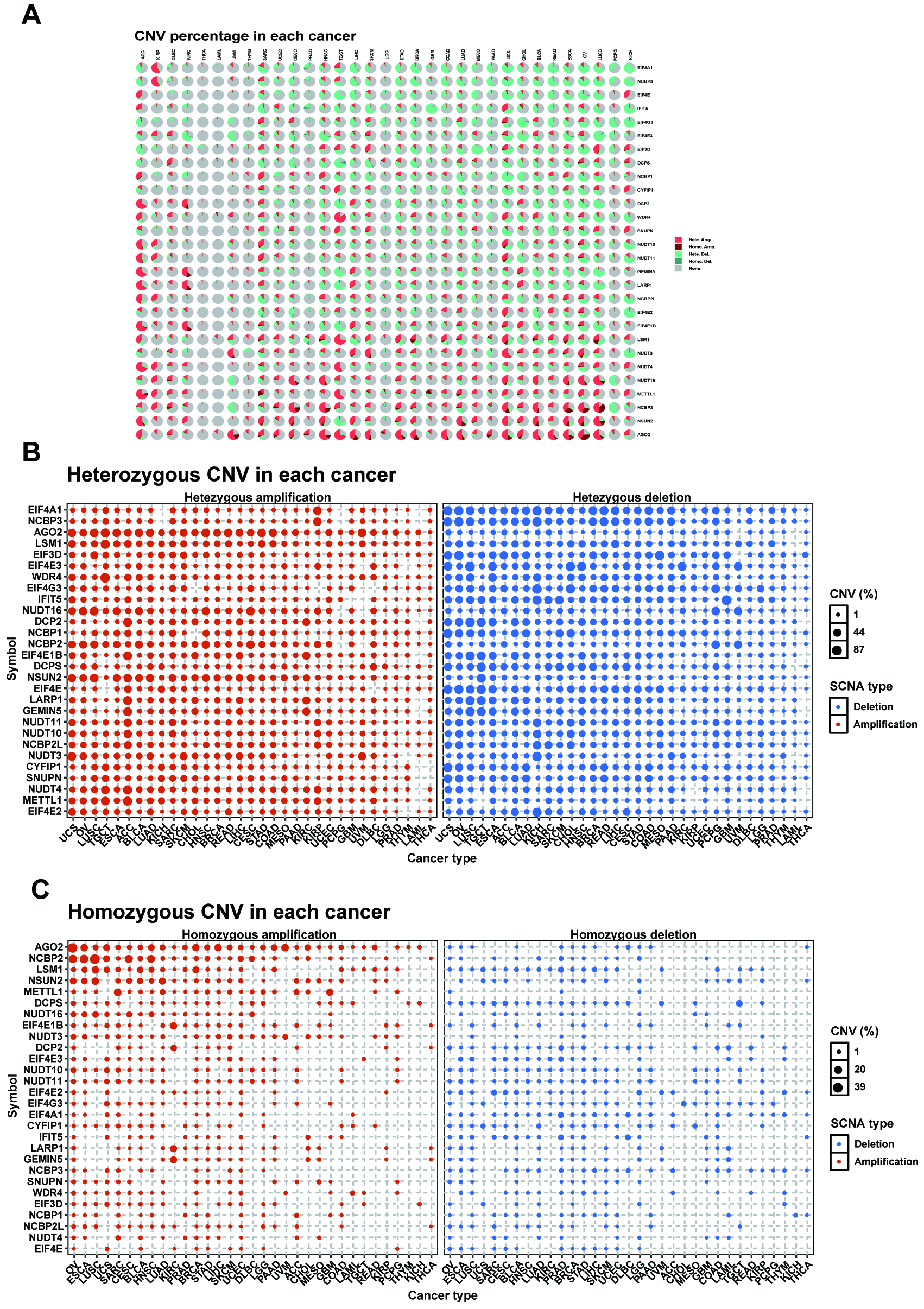

Supplement: Supplementary file 5 [file Image1.TIF]
