# Supplementary material for: Molecular characterization, clinical relevance and immune feature of m7G regulator genes across 33 cancer types
Source: Front Genet. 2022 Aug 25;13:981567. doi: 10.3389/fgene.2022.981567 (PMC9453236; doi:10.3389/fgene.2022.981567)
Supplement: Supplementary file 6 [file Table3.DOCX]

Supplementary Material

# Supplementary Figures and Tables

## Supplementary Figures

**Supplementary Figure 1.** Copy number variation of m7G RNA modification-related regulator genes. (A) CNV pie chart of 33 cancers. (B) Heterozygous CNV profile showed the percentage of amplification and deletion for regulator genes in cancers. (C) Homozygous CNV profile showed the percentage of amplification and deletion for regulator genes in cancers.

**Supplementary Figure 2.** Correlation of m7G modification level and m6A modification-related regulator gene expressions.

**Supplementary Figure 3.** Significant correlation of m7G modification level and OS in 7 cancers. (A) BRCA. (B) HNSC. (C) KIRC. (D) PAAD. (E) PCPG. (F) READ. (G) SARC.

**Supplementary Figure 4.** Significant correlation of m7G modification level and DFI, DSS, PFI cancers (p<0.05). (A-E) Association of m7G score to DFI in HNSC, LGG, PCPG, PADD and STAD. (F) Association of m7G score to DSS in SARC. (G-K) Association of m7G score to PFI in COAD, LIHC, BLCA, PAAD, PCPG.

## Supplementary Tables

**Supplementary Table 1.** List of 7G RNA modification-related regulator genes.

**Supplementary Table 2.** List of 33 cancer types.
